# Development and external validation of a prognostic nomogram for gastric cancer using the national cancer registry

**DOI:** 10.18632/oncotarget.8221

**Published:** 2016-03-21

**Authors:** Jianjun Liu, Qirong Geng, Zhimin Liu, Shangxiang Chen, Jing Guo, Pengfei Kong, YingBo Chen, Wei Li, Zhiwei Zhou, Xiaowei Sun, Youqing Zhan, Dazhi Xu

**Affiliations:** ^1^ State Key Laboratory of Oncology in South China, Collaborative Innovation Center for Cancer Medicine, Guangzhou, China; ^2^ Department of Gastric and Pancreatic Surgery, Sun Yat-sen University Cancer Center, Guangzhou, China; ^3^ Department of Hematology Oncology, Sun Yat-sen University Cancer Center, Guangzhou, China

**Keywords:** gastric cancer, nomogram, disease specific survival, SEER, prognosis

## Abstract

A nomogram based on both western and eastern populations to estimate the Disease Specific Survival (DSS) of resectable gastric cancer (RGC) has not been established. In current study, we retrospectively analyzed 4,379 RGC patients who underwent curative resection from the Surveillance, Epidemiology, and End Results (SEER) database. Patients diagnosed between 1998 and 2009 were assigned as training set (n= 2,770), and the rest were selected as SEER validation set (n= 1,609). An external validation was performed by a set of independent 1,358 RGC patients after D2 resection from Sun Yat–sen University Cancer Center (SYSUCC) in China. The nomogram was constructed based on the training set. The multivariate analysis identified that patient's age at diagnosis, race, tumor location, grade, depth of invasion, metastatic lymph node stage (mLNS) and total number of examined lymph node (TLN) were associated with patient's DSS. The discrimination of this nomogram was superior to that of the 7th edition of AJCC staging system in SEER validation set and SYSUCC validation set (0.73 versus 0.70, p=0.005; 0.76 versus 0.72, p=0.005; respectively). Calibration plots of the nomogram showed that the probability of DSS corresponded to actual observation closely. In conclusion, our nomogram resulted in more–reliable prognostic prediction for RGC patients in general population.

## INTRODUCTION

Although the incidence has declined recently, gastric cancer is still the second leading cause of cancer related–death and the 5–year survival was less than 30% [1]. Radical resection with D2 lymphadenectomy is the only potential curative method for RGC. However, the survival of RGC patients after D2 resection varies greatly due to different clinical pathological characteristics [2].

Currently, the American Joint Committee on Cancer (AJCC) classifies gastric cancer into nine groups in the 7th edition staging system [3]. This system assesses gastric cancer based on the depth of invasion, mLNS and the status of distant metastasis, and implies that the anatomical disease progression correlates with patients' survival. It has been widely used to predict the survival for gastric cancer patients. However, the variation of outcomes in intrastage patients cannot be accurately predicted by this staging system [4], especially the individual survival for each patient. It is believed that host status and other prognostic factors such as age, race and histology could significantly affect the individual survival in some cancers [5–9].

Nomogram, a simple statistical predictive tool, has been constructed in gastric cancer previously and proved to be useful and effective [10–17]. By creating an intuitive graph, a nomogram can predict a numerical probability of a special clinical event, such as overall survival (OS), progression–free survival and time to recurrence [18]. As nomograms based on single population might be unapplicable to RGC patients of all regions, it is of importance that nomograms be validated in multi–population cohort before clinical application [4]. However, only a few nomograms predicting survival probability of RGC patients were validated in different populations [10, 12, 19–21].

In the present study, we aim to develop and validate a nomogram for RGC based on a multi–institution and multi–population data from SEER database which contains both western and eastern patients with RGC. Additionally, we used a separate cohort from Asia for external validation.

## RESULTS

### Patients and demographics

4,379 gastric cancer patients from the SEER database between January, 2004 and December, 2012 were eligible for the present analysis (Table 1). Overall, the median age in the primary cohort was 64.3. The most common tumor sites were cardia and antrum (35.3%, 31.5% respectively). There were 973 (22.2%) Asian or Pacific Islander (API) patients and 3,406 (77.8%) nonAPI patients. The median follow-up was 28.5 months, and the 5–year DSS was 46.6%. 2,056 (46.9%) patients died before the analysis of the present study. The 2,770 patients diagnosed between 2004 and 2009 were assigned as training set, and patient's clinical pathological characteristics were listed in Table 2.

**Table 1:** Characteristic of primary cohort from SEER database

Characteristic	Patients(n= 4,379)		
	NO.		%
Age (years)			
Median		64.3±13.2	
Range		14 to 96	
Sex			
Male	2762		63.1
Female	1617		36.9
Race			
API	973		22.2
nonAPI	3406		77.8
Tumor size (cm) (n= 3,944)			
Median		5.6±6.5	
Range		0.1 to 9.5	
Tumor location			
Cardia	1544		35.3
Fundus	192		4.4
Body	562		12.8
Antrum	1379		31.5
Pylorus	204		4.7
Overlapping	498		11.4
Grade			
Well differentiated	178		4.1
Moderately differentiated	1126		25.7
Poorly differentiated	2941		67.2
Undifferentiated	134		3.1
Depth of invasion			
Mucosa or submucosa	777		17.7
Proper muscle	520		11.9
Subserosa	1748		39.9
Serosa	1002		22.9
Adjacent invasion	332		7.6
Number of positive LN			
0	1438		32.8
1 to 2	672		15.3
3 to 6	748		17.1
7 to 15	938		21.4
16 or more	583		13.3
Positive LN (Mean±SD)		6.4±8.4	
Total LN (Mean±SD)		26.5±11.2	
AJCC Stage			
IA	587		13.4
IB	346		7.9
IIA	599		13.7
IIB	558		12.7
IIIA	556		12.7
IIIB	913		20.8
IIIC	820		18.7
Combined devisceration			
Yes	658		15.0
No	3721		85.0

Abbreviation: API, Asian or Pacific Islander; LN, lymph node; AJCC, American Joint Committee on Cancer.

**Table 2:** Characteristics and multivariate analysis of the training set

	Characteristics		Multivariate Analysis		
	NO.	%	HR	95% CI	*p*
Age(Mean±SD year)	64.4±13.2	Range: 14 to 96	1.018	1.014 to 1.022	<0.001
Race					<0.001
API	613	22.1	1.262	1.101 to 1.447	
nonAPI	2157	77.9			
Location					<0.001
Antrum/Pylorus	997	36.0	ref		
Body	352	12.7	0.961	0.799 to1.156	
Cardia/Fundus	1111	40.1	1.306	1.153 to 1.479	
Overlapping	310	11.2	1.055	0.888 to 1.253	
Grade					0.002
Well differentiated	89	3.2	ref		
Moderately differentiated	696	25.1	1.358	0.870 to 2.121	
Poorly differentiated	1894	68.4	1.697	1.095 to 2.628	
Undifferentiated	91	3.3	1.713	1.028 to 2.854	
Total LN (Mean±SD)	26.4±11.2		0.985	0.980 to 0.990	<0.001
Depth of invasion					<0.001
Mucosa or submucosa	444	16.0	ref		
Proper muscle	314	11.3	1.502	1.094 to 2.061	
Subserosa	1058	38.2	2.844	2.190 to 3.693	
Serosa	713	25.7	3.155	2.411 to 4.127	
Adjacent invasion	241	8.7	4.387	3.269 to 5.887	
Number of positive LN.					<0.001
0	841	30.4	ref		
1 to 2	417	15.1	1.729	1.410 to 2.118	
3 to 6	456	16.5	2.221	1.830 to 2.696	
7 to 15	644	23.2	3.220	2.683 to 3.864	
16 or more	412	14.9	6.126	5.018 to 7.478	

Abbreviation: API, Asian or Pacific Islander; LN, lymph node;HR:hazard ratio;

There were two external validation sets used to validate the nomogram in the present analysis. 1,609 gastric cancer patients diagnosed between 2010 and 2012 from SEER data were selected as SEER validation set. 1,385 RGC patients underwent D2 resection in SYSUCC from 2000 to 2011 were assigned as SYSUCC validation set. The clinical pathological characteristics were listed in Table 3.

**Table 3:** Characteristic of validation sets

	SEER-Validation set(n=1,609)			SYSUCC-Validation set(n=1,385)		
	NO.		%	NO.		%
Age (years)						
Median	64.0±13.2			56.6±12.1		
Range	22 to 94			16 to 89		
Sex						
Male	1028		63.9	926		66.9
Female	581		36.1	459		33.1
Race						
API	360		22.4	1385		100
nonAPI	1249		77.6			
Tumor location						
Antrum/Pylorus	586		36.4	601		43.4
Body	210		13.1	262		18.9
Cardia/Fundus	625		38.8	522		37.7
Overlapping	188		11.7			
Grade						
Well differentiated	89		5.5	15		1.1
Moderately differentiated	430		26.7	366		26.4
Poorly differentiated	1047		65.1	997		72.0
Undifferentiated	43		2.7	7		0.5
Depth of invasion						
Mucosa or submucosa	333		20.7	147		10.6
Proper muscle	206		12.8	162		11.7
Subserosa	690		42.9	370		26.7
Serosa	289		18.0	574		41.4
Adjacent invasion	91		5.7	132		9.5
Number of positive LN.						
0	597		37.1	331		23.9
1 to 2	255		15.8	235		17.0
3 to 6	292		18.1	247		17.8
7 or 15	294		18.3	355		25.6
16 or more	171		10.6	217		15.7
No. of Positive LN (Mean±SD)	5.4±7.8			7.5±8.9		
Total LN (Mean±SD)	26.6±11.2			28.7±10.1		
AJCC Stage						
IA	256		15.9	103		7.4
IB	143		809	94		6.8
IIA	264		16.4	120		8.7
IIB	212		13.2	196		14.2
IIIA	206		12.8	163		11.8
IIIB	283		17.6	283		20.4
IIIC	245		15.2	426		30.8

Abbreviation: API, Asian or Pacific Islander; LN, lymph node; AJCC, American Joint Committee on Cancer.

### Selected independent risk factors for the nomogram construction

**Figure 1:**
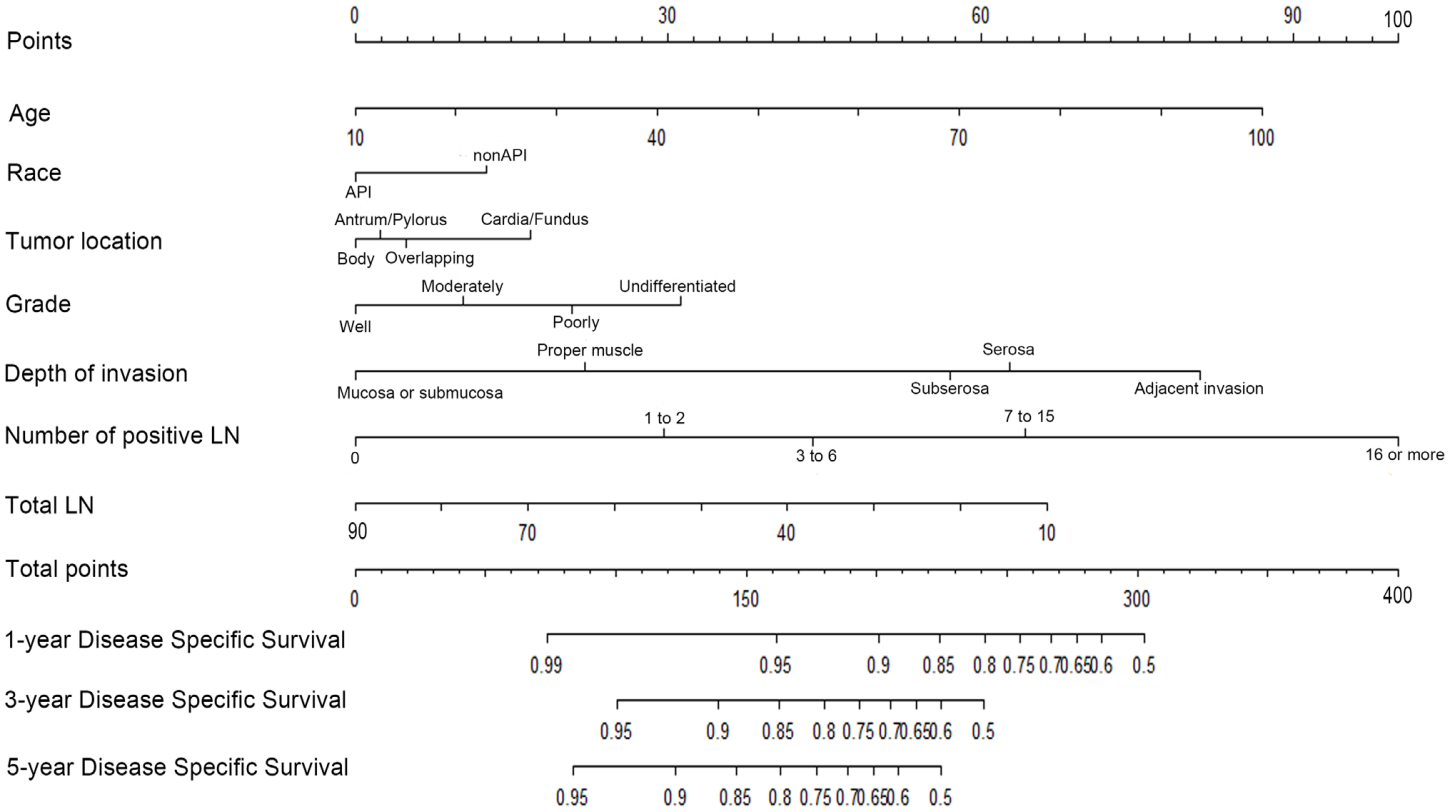
Nomogram predicting 1–year, 3–year and 5–year DSS for RGC patients after curative resection The nomogram is used by adding up the points identified on the points scale for each variable. According to the sum of these points projected on the bottom scales, the nomogram can provide the likelihood of 1–year, 3–year and 5–year DSS for an individual patient.

Clinical pathological variables were transformed and examined to fit the Cox PH regression and linear assumption before models construction. The potential variables from training set were analyzed by the forward method in multivariate analysis. As listed in the Table 2, the patients' age at diagnosis, race, tumor location, grade, depth of invasion, mLNS and TLN were associated with patients' DSS (Chi–square test=1068.9, p<0.001), and the nomogram was constructed from this model (Figure 1).

### Validation of the nomogram

The external validation of the nomogram was performed by two individual external validation sets (SEER validation set and SYSUCC validation set). The clinical pathological characteristics of validation sets were listed in Table 3. The predictive ability of the nomogram was compared to the 7th edition of AJCC staging system. First, the nomogram was validated by the SEER validation set. The C–index of which was obviously higher than that of the 7th edition of AJCC staging system (0.73, 95% CI, 0.70–0.76 versus 0.70, 95% CI, 0.67–0.74; p=0.005). Second, the discrimination of the nomogram was evaluated by the SYSUCC validation set. Interestingly, the nomogram based on the western (including 77.8% nonAPI) population also has an optimal discrimination in Asian population (C–index of nomogram: 0.76, 95%CI, 0.73–0.78 versus C–index of 7th edition of AJCC staging system: 0.72, 95%, 0.69–0.74; p= 0.005).

**Figure 2:**
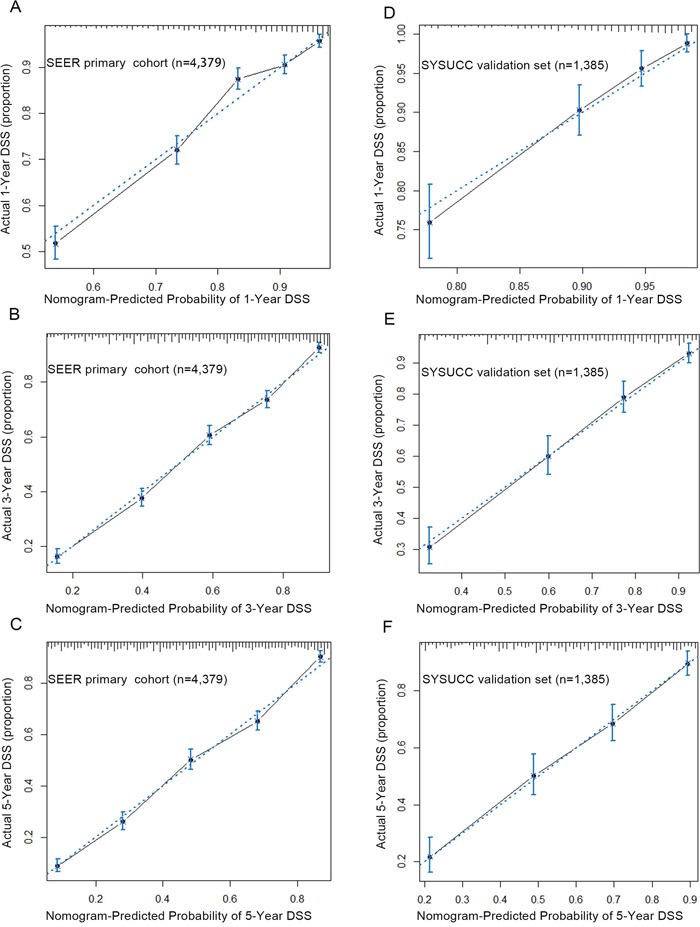
The calibration curve for predicting patients' DSS at 1–year A. 3–year B. and 5–year C. in the SEER primary cohort and predicting DSS at 1–year D. 3–year E. and 5–year F. in the SYSUCC validation set The X–aixs represents the nomogram–predicted survival, and the actual survival is plotted on the Y–axis. The dotted line represents the ideal correlationship between predicted and actual survival. Abbreviation: SEER, the Surveillance, Epidemiology, and End Results cancer registries; SYSUCC, Sun Yat–sen University Cancer Center; DSS, Disease Specific Survival.

**Figure 3:**
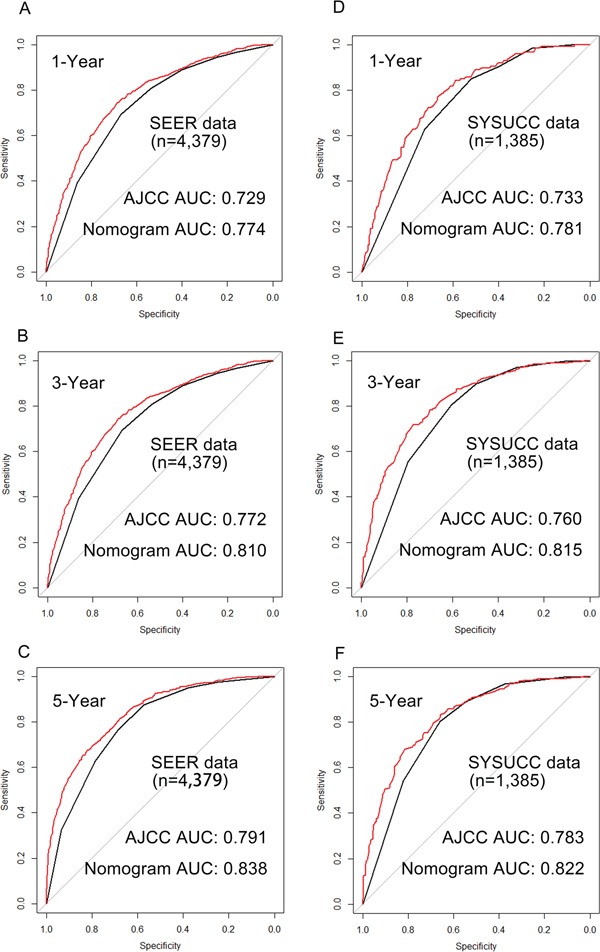
Comparison of the areas under the receiver operating curves of nomogram and AJCC to prediction of DSS at 1–year A. 3–year B. and 5–year C. in the SEER primary cohort and 1–year D. 3–year E. and 5–year F. in the SYSUCC validation set The red lines represent nomogram predicted DSS and the balack lines represent the AJCC staging predicted DSS. Abbreviation: SEER, the Surveillance, Epidemiology, and End Results cancer registries; SYSUCC, Sun Yat–sen University Cancer Center; DSS, Disease Specific Survival.

Next, considering that the longest follow–up of SEER validation set was 35 months, the 5–year calibration cannot be executed in SEER validation set. Therefore, the calibration plots were separately performed by the primary cohort and SYSUCC validation set. As shown in Figure 2, calibration plots show that the predicted 1–year, 3–year and 5–year DSS corresponded closely to the actual survival estimated by the Kaplan–Meier method in the two data sets. Additionally, we compared the 1–year, 3–year and 5–year DSS predicting ability of the two models by the AUC (area of ROC curve) in the two data sets (Figure 3). As shown in the Figure 3 and Table 4, the nomogram shows superior survival predictive ability than the 7th AJCC staging system.

**Table 4:** Comparison of the areas under the ROC curves for nomogram and the 7th edition of AJCC staging system in each time points

Time points	Nomogram		AJCC staging system		p
	AUC	95%CI	AUC	95%CI	
SEER primary cohort					
1-Year	0.774	0.756 to 0.792	0.729	0.710 to 0.747	<0.001
3-Year	0.810	0.795 to 0.826	0.772	0.755 to 0.790	<0.001
5-Year	0.838	0.820 to 0.856	0.791	0.769 to 0.813	<0.001
SYSUCC validation set					
1-Year	0.781	0.742 to 0.820	0.733	0.694 to 0.772	0.001
3-Year	0.815	0.786 to 0.843	0.760	0.728 to 0.792	<0.001
5-Year	0.822	0.790 to 0.855	0.783	0.745 to 0.821	<0.001

Abbreviation: AUC, Area Under the ROC Curve; AJCC, American Joint Committee on Cancer.

## DISCUSSION

In this study, we developed and validated a novel nomogram of RGC patients underwent curative resection to predict DSS based on general population. A total of 4,379 gastric patients from SEER database and 1,385 RGC patients from Asia were analyzed. Our nomogram showed better predictive accuracy than the 7th edition of AJCC staging system in DSS prediction for the RGC patients (C–index: 0.73 versus 0.70, p=0.005 in SEER validation set; 0.76 versus 0.72, p=0.005 in SYSUCC validation set; respectively).

Several nomograms have been constructed in RGC patients, and show more accurate survival prediction than the conventional staging system in different populations. In 2003, Kattan et al developed a nomogram to predict 5–year DSS for gastric cancer patients based on 1,136 patients from Memorial Sloan–Kettering Cancer Center (MSKCC), and Han et al developed and validated a nomogram in a cohort of 10,454 gastric cancer patients who underwent curative resection form Seoul National University Hospital (SNUH, Seoul, Korea) and Cancer Institute Ariake Hospital (Tokyo, Japan) in 2012 [19, 20]. Both MSKCC nomogram and SNUH nomogram showed that combining more clinical pathological characteristics can provide an improved accuracy for survival prediction (0.80 versus 0.77, p<0.001; 0.78, 0.79 versus 0.69; respectively). However, the MSKCC nomogram was validated by an internal validation (bootstrap resampling), and the SNUH nomogram was developed and validated only in Asian region. Thus, it's unclear whether it is applicable for the general population. Actually, external validation of the nomogram is essential. This process can test the bias of the estimation of nomogram performance in different populations and judge the applicability to other different populations [18].

Compared with previous MSKCC nomogram and SNUH nomogram, our nomogram was developed and validated based both on western and eastern population. In this study, the race of patients was categorized as API and nonAPI. Interestingly, on the multivariate analysis, we found the nonAPI patients had a worse prognosis than API patients in the SEER data (hazard ratio: 1.337, p<0.001), which was consistent with previous studies [6, 7, 9]. Indeed, even in the same TNM stage, patients from different populations might lead to various survival, the reason may be the missing prognostic factor, the race. Currently, our nomogram was first time to use the patient's race as one of risk factors and could predict the DSS in general population more precisely.

Improving the accuracy of the survival estimation is exceedingly important for clinical decision. There are several advantages by using nomogram. Firstly, the accurate prediction would be favor for designing postoperative treatment. For example, in 2010, a phase III trial confirmed that adjuvant chemotherapy with S1 (an oral fluoropyrimidine) was an effective treatment for advanced gastric cancer patients who underwent D2 gastrectomy [22]. However, it is still uncertain whether all the RGC patients, especially, the patients with better prognosis require adjuvant chemotherapy. Since our nomogram could make a more accurate prediction of individual survival than 7th edition of AJCC staging system, it may be an effective criterion for patients to design an individual postoperative treatment. Secondly, our nomogram can calculate each patient's 1–year, 3–year and 5–year survival rate respectively. Therefore, it has potential to be used for a more reasonable follow–up schedule. Thirdly, nomogram can be used for patients' consultant. The variation of DSS intrastage can't be predicted accurately by traditional TNM–stage system. By contrast, our nomogram can provide individualized estimation for gastric cancer patients.

There are some limitations should be acknowledged. Firstly, only the patients who had complete information were included in present study, there may be a selection bias. Secondly, as this nomogram was based on SEER database, analysis was limited to the prognosis factors in the database. Several predictors such as Lauren classification, genetic differences, protein expression differences and postoperative treatments had not been included [23–25].

In summary, we first develop and validate a prognostic nomogram based on a multi–institution and multi–population database predicting short–term and long–term DSS for RGC patients. Compared with the 7th edition of AJCC staging system, the proposed nomogram represents better prognostic discrimination and predictive accuracy for DSS. It can be used to calculate individualized survival prediction and provide better treatment allocation after curative resection.

## PATIENTS AND METHODS

### Patients

The SEER program is a national collaboration program by the National Cancer Institute. It collects and publishes approximately 3 million cases from a variety of geographic regions and covers 26% American population's cancer incidence and survival data. A retrospective review of all gastric cancer patients underwent gastrectomy from SEER database between 1998 and 2012 was performed. A total of 31,988 cases from SEER 18 registries were initially screened. Patients were excluded if they had incomplete information on depth of invasion, tumor size, positive lymph node (PLN), TLN or status of distant metastasis. Given that the 7th edition of AJCC staging system bases mLNS definition on the absolute PLN and suggests that “at least 16 regional lymph node be assessed pathologically”, the patients (n=8,107) with TLN less than 16 were excluded from present study [3]. The remaining (n= 4,379) were defined as SEER primary cohort. Based on the SEER primary cohort, patients diagnosed as gastric cancer between 1998 and 2009 were assigned as training data set, and those between 2010 and 2012 were SEER validation set.

The proposed nomogram was also externally validated by SYSUCC validation set. Of the 2,205 RGC patients who underwent D2 resection in SYSUCC between 2000 and 2011, 1,385 patients met the following inclusion criteria: no history of receiving anti–cancer therapy before surgery; no history of other malignancies; no distant metastasis; complete resection of cancer (R0 resection) with D2 lymphadenectomy; number of examined lymph more than 15; without one or more missing characteristics. The median follow-up was 36.8 months in training set, 14.0 months in SEER validation set and 36.7 months in SYSUCC validation set.

### Study design

The data of patients' clinicopathological characteristics such as age at diagnosis, sex, race, surgery, tumor location, size, histology, grade, depth of invasion, PLN and TLN were collected. The pathological tumor stage, depth of invasion and mLNS were restaged according to the 7th edition of AJCC staging system [3]. The primary endpoint was DSS, which was defined as the time form surgery to cancer–related death or the last follow–up. The follow–up duration was measured as the time from the date of surgery to the last follow–up. The survival status was recorded according to the latest follow–up.

### Construction of the nomogram

Based on clinical findings, categorical variables were grouped before modeling. Restricted cubic splines were used to evaluate the linear relationship between continuous variables and DSS [19]. Continuous variables were transformed into categorical variables to fit the linear assumption [20]. Independent risk factors were identified by the forward stepwise in the Cox proportional hazards (PH) regression model. DSS estimation and survival curves were performed by Kaplan–Meier method and validated by the log–rank test.

Nomogram was established based on the training set data. Based on the results of Cox PH regression, a nomogram combining all the independent prognostic factors was constructed for 1–year, 3–year and 5–year DSS predicting by using the package of rms in R software version 3.1.3 (http://www.r-project.org/).

### Validation of the nomogram

The nomogram was validated by measuring both discrimination and calibration using two separated data sets. Firstly, the discrimination of nomogram was evaluated by Harrell's C–index, which can estimate the probability between the observed and predicted DSS. The higher the C–index, the more precise the survival prediction was. Discrimination between the proposed nomogram and the 7th edition of AJCC staging system was performed by the roccp. cens package in R. Following, calibration were carried out by grouping all the patients firstly, and then the mean of the groups were compared with observed Kaplan–Meier DSS estimation. Finally, the precision of survival prediction in 1–year, 3–year and 5–year time points were evaluated by the area under receiver operating characteristic (ROC) curve.

p<0.05 will be considered as statistically significant. All statistics analysis were performed by the R software version 3.13 (http://www.r-project.org/) and the software statistical package for social sciences version 19.0 (SPSS, Chicago, IL).
